# In Silico-Identified miR-16-5p and miR-32-5p as a Shared Molecular Signature of Primary Gliomas and Parkinson’s Disease: Plasma Levels Are Increased Only in Glioma Patients

**DOI:** 10.3390/brainsci16040347

**Published:** 2026-03-24

**Authors:** Janusz Szyndler, Zofia Wicik, Anna Wierucka, Piotr Maciejak, Michał Sobstyl, Angelika Stapińska-Syniec, Piotr Glinka, Karol Piwowarski, Natalia Chmielewska

**Affiliations:** 1 Department of Experimental and Clinical Pharmacology, Center for Preclinical Research and Technology CePT, Medical University of Warsaw, Banacha 1B Street, 02-097 Warsaw, Poland; janusz.szyndler@wum.edu.pl; 2 Department of Experimental and Clinical Neuroscience, Institute of Psychiatry and Neurology, Sobieskiego 9 Street, 02-957 Warsaw, Poland; zwicik@ipin.edu.pl (Z.W.); pmaciejak@ipin.edu.pl (P.M.); 3 Faculty of Medicine, Medical University of Warsaw, Zwirki and Wigury 61 Street, 02-097 Warsaw, Poland; s083272@student.wum.edu.pl; 4 Department of Neurosurgery, Institute of Psychiatry and Neurology, Warsaw, Poland, Sobieskiego 9 Street 02-957 Warsaw, Poland; msobstyl@ipin.edu.pl (M.S.); astapinska@ipin.edu.pl (A.S.-S.); pglinka@ipin.edu.pl (P.G.); kpiwowarski@ipin.edu.pl (K.P.)

**Keywords:** Parkinson’s disease, gliomas, miR-16-5p, miR-32-5p, p53

## Abstract

**Highlights:**

**What are the main findings?**

- A 953 of genes was shared between glioblastoma and Parkinson’s disease (PD), indicating substantial overlap in disease-related molecular pathways.
- Plasma levels of miR-16-5p and miR-32-5p were significantly higher in glioma patients compared with PD patients and controls, and miR-16-5p together with p53 were increased in glioblastoma tumor tissue.

**What are the implications of the main findings?**

- miR-16-5p and miR-32-5p may serve as peripheral biomarkers associated with glioma-related molecular pathways.
- The shared gene network indicates a potential molecular connection between neurodegeneration and glioma biology. Further studies are needed to identify specific pathways involved in both cell death and proliferation.

**Abstract:**

Objectives: In this study, we explore the molecular basis of the literature-reported inverse association between brain neoplasms and neurodegenerative disorders, including Parkinson’s disease (PD). As miRNAs are post-transcriptional regulators, we selected them as candidates underlying opposite processes of neurodegeneration and glioma development. Methods: We used bioinformatic analyses for disease-gene extraction, miRNA target prediction, enrichment analyses, and miRNA ranking. We identified 953 shared genes between PD and glioblastoma (GBM) in DisGeNET, then prioritized miRNAs predicted to regulate the largest number of shared targets. Next, we collected peripheral blood from patients with PD (*n* = 12), patients with gliomas (the most advanced—grade IV, *n* = 10 and grade III *n* = 3) and controls undergoing spinal surgery for disk pathology (*n* = 10). Blood samples were obtained pre-operatively in controls and glioma patients. Tumor and peritumoral tissues were obtained from glioma patients, whereas tissue sampling is not feasible in PD. Brain tissues and plasma were analyzed using RT-qPCR (miRNA) and ELISA (p53). Results: We observed increased levels of miR-16-5p (*p* < 0.05) and p53 protein (*p* < 0.05) in tumor tissues compared with peritumoral tissue. Additionally, miR-16-5p and miR-32-5p plasma levels were elevated in glioma patients compared with both PD patients (*p* < 0.01 and *p* < 0.001, respectively) and controls (*p* < 0.01 and *p* < 0.001, respectively). Plasma levels in PD did not differ from controls. Conclusions: Although these analyses highlight miR-16-5p and miR-32-5p as candidate biomarkers associated with glioma related pathways, the results did not provide evidence for the expected opposite regulation between PD and glioma. Future studies with a larger cohort of patients using high-throughput methods are needed to validate these findings and to elucidate the mechanisms driving neurodegeneration or excessive proliferation.

## 1. Introduction

Brain and other central nervous system (CNS) tumors, although rare, contribute significantly to morbidity and mortality across all age groups [1]. Gliomas are the most common type of primary intracranial tumor, accounting for 81% of malignant brain tumors [2]. Among gliomas, glioblastoma is the most prevalent, comprising 45% of all gliomas. It is also the most common primary malignant brain tumor, with an incidence rate of 3.23 cases per 100,000 people [3]. Survival rates vary significantly depending on the tumor type. The five-year survival rate for pilocytic astrocytoma is 94.7%, while for glioblastoma, it is 6.8% [4]. Together, these data underscore the high unmet need for improved diagnostic and prognostic tools in malignant gliomas.

Parkinson’s disease (PD) is one of the most prevalent neurodegenerative movement disorders [5]. In Europe, the prevalence and incidence of PD are estimated to be approximately 108–257 per 100,000 and 11–19 per 100,000 per year, respectively [6]. The treatment of PD is primarily symptomatic, focusing on the dopaminergic pathway. Currently, no disease-modifying therapies are available for PD. The disease is associated with an increased risk of all-cause mortality, a reduction in life expectancy, and a high risk of severe disability [6]. Therefore, identifying molecular pathways relevant to PD pathophysiology and progression remains a key research priority.

Despite the divergent molecular etiopathology of gliomas and PD, current epidemiological data suggest a relationship between their pathophysiology. Increasing epidemiological evidence suggests an inverse association between the occurrence of neurodegenerative diseases and cancer [7,8,9,10,11].

Although many epidemiological studies have demonstrated this inverse correlation, the mechanistic basis of this phenomenon remains poorly understood [12,13,14]. However, a strong association with cell cycle pathways is suggested, given the significant neuronal loss in neurodegenerative diseases and uncontrolled cell proliferation in tumors [7,8,9,10,11]. It has been proposed that dysregulation of certain genes, particularly those involved in cell cycle regulation, may simultaneously increase the risk of neurodegeneration and reduce cancer risk, or conversely, decrease neurodegeneration risk while increasing cancer susceptibility [15]. This concept motivates the search for molecular regulators that intersect neurodegeneration- and tumor-related pathways.

In recent years, significant advances have been made in identifying miRNA candidates with potential as peripheral biomarkers of glioblastoma [16,17]. Circulating miRNAs play key roles in cancer progression and hold promise as biomarkers for the diagnosis, prognosis and treatment monitoring of glioblastoma. A systematic review and meta-analysis demonstrated that microRNAs exhibit high diagnostic accuracy for glioma, with a pooled Area Under the Curve (AUC) of 0.893, underscoring their potential as non-invasive biomarkers for both diagnosis and prognosis, although specific miRNAs were not individually identified [16]. Moreover, circulating miR-29a, miR-106a, and miR-200a were found to be upregulated in the blood of glioblastoma patients and downregulated following therapeutic interventions such as surgery, chemo-therapy, or radiation therapy [17]. However, despite advances in the field, validated biomarkers that facilitate diagnosis and track treatment progression in gliomas are still lacking. Identifying a peripheral biomarker, particularly one mechanistically linked to etiopathogenesis, holds significant potential. Such biomarkers may complement current diagnostic and monitoring approaches, although clinical translation will require robust validation.

Beyond their diagnostic potential, miRNAs may also play a role in disease etiopathology. It is noteworthy that RNA-based therapeutic technologies, such as inclisiran for hypercholesterolemia and givosiran for porphyria, have already been approved for clinical use [18,19]. These achievements indicate the possibility of using miRNA-targeting strategies, including those involving miRNA pathways, as therapeutic targets in the future. Nonetheless, miRNA-based therapeutics face substantial challenges related to specificity, delivery and safety.

Given the complexity of miRNA biology and the extensive transcriptional alterations accompanying glioma development, it is reasonable to consider that not a single miRNA, but rather combinations or panels of miRNAs, may emerge as highly promising diagnostic indicators in clinical medicine. Moreover, identifying molecules that are simultaneously involved in both glioma and PD pathways may, in the future, help elucidate specific mechanisms underlying the transition of healthy brain tissue into either neurodegenerative or tumorigenic states [7,8,9,10,11], thereby contributing to the development of novel therapeutic strategies targeting these shared molecular pathways.

In this study, we integrate gene sets associated with PD and glioblastoma to identify shared pathways and priority candidate regulatory miRNAs rather than to establish causal mechanisms. Unlike many prior analyses that focus on individual diseases or on differential expression alone [20,21,22], we employ a multi-layered systems biology approach that combines curated gene–disease associations from DisGeNET with miRNA target prediction, enrichment analysis and network visualization. By analyzing the interaction networks of miR-16-5p and miR-32-5p, two prioritized candidate miRNAs predicted to regulate genes implicated in both diseases, together with their expression in plasma and tumor/peritumoral tissue, we generate hypotheses about converging pathways at the interface of neurodegeneration and tumorigenesis. Enrichment analyses and network-based visualization were used to highlight biological themes potentially associated with the prioritized candidates, providing an integrative view of their putative functional context in glioma and PD in a small, well-defined patient cohort. In addition to miRNA candidates, p53 was included as a complementary marker because it represents a major regulatory node linking cell-cycle arrest, apoptosis, and cellular stress responses relevant to both tumorigenesis and neurodegeneration. Accordingly, p53 was analyzed not as an independent primary endpoint, but as a complementary marker to support interpretation of the selected miRNA findings.

## 2. Methods

### 2.1. Bioinformatic Assessment

Gene lists extraction. To identify genes and then miRNAs regulating processes shared between PD and glioblastoma we downloaded from the DisGeNET database lists of genes associated with 28 PD related terms (including 2177 genes) and 14 glioblastoma related terms (including 3377 genes). Shared genes were defined as the intersection between the PD-associated and glioblastoma-associated gene lists, resulting in 953 genes. We included only human gene–disease associations reported in curated and literature-based sources in DisGeNET (Barcelona, Spain), recognizing that these encompass evidence of varying strength. DisGeNET-derived shared genes were therefore used as an initial screening layer for downstream miRNA prediction, ranking and enrichment analyses.

miRNA target prediction. After identification of PD-related, glioblastoma-related and shared genes we screened for miRNAs targeting those genes using 14 prediction data-bases implemented in the multimiR R package [23]. A variety of tools exist for miRNA target prediction, including miRDB, machine-learning approaches such as mintRULS, and integrative frameworks like PRIMITI, which combine sequence features with ex-pression data. These methods differ in the underlying training data and scoring schemes, and we believe that no single resource is universally superior. In the present study we used the multiMiR R package because it aggregates predicted and validated miRNA–target interactions from multiple databases within a single workflow, allowing us to capture consensus signals across tools rather than depending on a single predictor.

This approach allowed us to collect predicted miRNA–target interactions from multiple resources within a single workflow. For each candidate miRNA, we counted the number of predicted targets among the shared PD–glioblastoma genes, as well as in the PD-only and glioblastoma-only gene sets. miRNAs were then ranked according to the number of predicted targets in the shared gene set, and the top 10% miRNAs by shared target count were considered for further prioritization. To aggregate and rank the data we used R package wizbionet developed by our bioinformatician, https://github.com/wizbionet/wizbionet/blob/master/doc/vignette_wizbionet.md (accessed on 5 May 2023) [24].

Ranking of the miRNAs. Top miRNAs were selected based on the number of predicted targets among the 953 shared PD–glioblastoma genes, and on the number of predicted targets in the PD-only and glioblastoma-only gene sets.

Enrichment analysis. To identify crucial processes and pathways shared between both diseases, enrichment analysis was performed using the EnrichR REST API from the R environment [25], with the Hypergeometric test with Benjamini and Hochberg correction, while the reference was the human genome. Next, we extracted gene lists of the top enriched terms (shared, PD- and glioblastoma-related), miR-16-5p and miR-32-5p targets. Enrichment analysis allowed us to identify the top 5 most significant ontological terms, for each database analyzed. In the main manuscript, we report only terms that remained significant after Benjamini–Hochberg correction (adjusted *p* < 0.05).

Interaction network construction. To identify top genes within interaction networks, we constructed an interaction network between miR-16-5p and miR-32-5p and their targets. To identify target–target interactions, we obtained data from the human interactome using StringApp for Cytoscape (version 1.5.1). Genes were grouped into those regulated by miR-16-5p, miR-32-5p and shared by both miRNAs. Further, we selected genes with the highest number of connections with other genes from a shared gene list: top 20 genes regulated by both miRNAs, and 15 genes for each miRNA. Visualization was performed in Cytoscape software using a degree-sorted circular layout.

### 2.2. Patients

**Figure 1:**
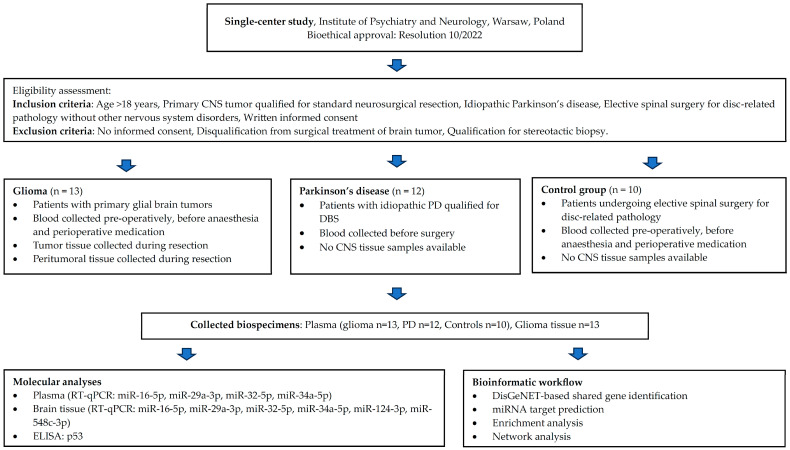
The flowchart of the study. The arrows illustrate the stepwise flow from patient enrollment through sample collection to molecular and computational analyses.

Patients included in the study were hospitalized in the Neurosurgical Department of the Institute of Psychiatry and Neurology in Warsaw, Poland (single-center), and all procedures were approved by the local Bioethical Commission (Resolution 10/2022) (see Figure 1, representing the flowchart of the presented study). The study material consisted of two groups of patients (one group of patients with primary glial brain tumors, *n* = 13, the other with long-standing neurodegenerative disorder—PD; *n* = 12) and the control group consisted of patients treated for elective spinal surgery for disk-related pathology without a history of brain tumors and neurodegenerative disorders (*n* = 10). Blood samples were collected from patients with primary glial cell tumors, patients with PD, and control groups. Blood was collected pre-operatively and prior to anesthesia and perioperative medication in the glioma and control groups. For PD patients, blood sampling was performed in the medication-ON state.

Biopsy samples, including tumor tissue and peri-tumoral margins, were collected from patients with gliomas during brain tumor resection procedures. The aim was to investigate potential correlations between the molecular changes in the peripheral tissues and the CNS.

Patients were operated on according to standard techniques utilized for different neurosurgical conditions. All patients signed written informed consent before all types of elective neurosurgical procedures. Moreover, patients were informed about the main concept of the present study and were fully informed about the planned study’s goals. Their participation in the study was voluntary, and no one from the medical staff convinced the patients to take part in this study. Refusal to participate in the study had no impact on the neurosurgical procedure performed.

Glioma group. The study cohort included 13 patients, comprising 7 females and 6 males, with an average age of 58.0 years (range: 38–76 years). The mean symptom duration till surgery was 2 weeks (range, 1–5 weeks). All patients had preoperative computed tomography (CT) and standard magnetic resonance imaging (MRI) with contrast enhancement. Based on preoperative MRI images, the suspicion of a glioma was raised. All patients after an anesthesiologist consultation had a standard craniotomy performed in general anesthesia with the aid of neuronavigation. All included patients had a postoperative scheduled CT to exclude any intracranial hematoma within 6 to 10 h after neurosurgical operation. According to the fifth edition of the World Health Organization (WHO) Classification of Tumors of the CNS, there were 6 patients with a diagnosis of IDH wildtype glioma, 4 patients with a diagnosis of IDH mutant glioma and 3 patients diagnosed with glioma NOS (not otherwise specified). For comparison with the previous WHO grading system, 10 patients were diagnosed with Grade IV (glioblastoma multiforme) and 3 patients with Grade III (anaplastic astrocytoma).

Parkinson’s disease group. The patients with PD recruited for this study were candidates for deep brain stimulation (DBS) procedures to ameliorate disabling motor symptoms in the medication-off state and suffered from severe levodopa-induced dyskinesia and off-on motor fluctuations in the medication-on state. The PD group comprised 12 patients, including 10 males and 2 females, with an average age of 67.2 years (range: 55–76 years). The average duration of disease was 12.25 years (range: 8–20 years). Disease severity was assessed using the Hoehn and Yahr (H&Y) scale and Unified Parkinson’s Disease Rating Scale (UPDRS). The mean H&Y score was 3.58 in the “off” state and improved to 2.00 in the “on” state after L-DOPA treatment. Similarly, the mean UPDRS III score improved from 54.83 in the “off” state to 25.5 in the “on” state. The Parkinson’s disease group demonstrated significant motor improvement with L-DOPA, but the advanced stage of disease progression was evident in their baseline motor impairments and residual symptoms after treatment. All stereotactic surgeries were done without surgery-related intraoperative complications. All patients after DBS leads implantation had intraprocedural CT to exclude hemorrhagic complications and to check the proper placement of DBS leads. After surgeries, the patients were discharged home on the third postoperative day. The scheduled follow-up visits took place at our ambulatory department to set the proper stimulation parameters.

Control group. The control group consisted of 10 patients without history of neoplasm or neurodegenerative disorder. The patients had radiculopathy compression syndromes in cervical (2 patients) or lumbar (8 patients) spine. All patients underwent standard disk herniation removal through cervical microdiscectomy with implant placement in cervical spine or standard lumbar discectomy using microneurosurgical technique. In the control group, there were 6 women and 4 men. The mean age at surgery was 49.3 years (range 37–76 years). All spinal surgeries were uneventful, and patients were discharged home except 1 patient who was sent to neurorehabilitation department due to preoperative paraparesis.

### 2.3. Plasma and Tissue Collection, Initial Processing and Total mRNA Isolation

Plasma was collected from all patients participating in the study: patients with primary glial brain tumors (*n* = 13), patients with long-term PD (*n* = 12), and patients treated for cervical or lumbar disk herniation syndromes without a history of brain tumors and neurodegenerative disorders (*n* = 10). Blood was obtained pre-operatively and before perioperative medications. Brain tissue, including tumoral and peritumoral samples, was collected only from patients with primary glial brain tumors (*n* = 13).

These plasma samples were subsequently used to quantify the expression of four miRNAs (miR-16-5p, miR-29a-3p, miR-32-5p and miR-34a-5p), whereas tumor and peritumoral tissue samples were used to measure six miRNAs (the above four miRNAs plus miR-124-3p and miR-548c-3p). The selection rationale for these miRNAs is described in detail in section “Bioinformatic Assessment”.

Venous blood samples were collected using S-Monovette^®^ K3 EDTA tubes (Sarstedt AG & Co. KG; Nümbrecht, Germany) and centrifuged at 2500× *g* for 15 min at 4 °C. Blood was processed fresh (without prior freezing) and centrifuged once immediately after collection. The resulting plasma samples were aliquoted and stored at −70 °C until further processing. Plasma was inspected visually for gross hemolysis prior to downstream analyses. Samples with visible hemolysis were not observed. To minimize cellular contamination, plasma was transferred to a new tube without disturbing the buffy coat.

Brain samples (tumor and peritumoral tissue), devoted to miRNA analysis, were collected during resection, placed in ice-cold 0.9% NaCl, and transferred to Allprotect Tissue Reagent (QIAGEN, Hilden, Germany) for immediate RNA stabilization, followed by storage at −70 °C. The part of the tissue devoted to enzyme-linked immunosorbent assay (ELISA) was immediately frozen after resection at −70 °C.

Plasma samples were subjected to miRNA isolation using the Invitrogen™ mir-Vana™ miRNA Isolation Kit (Thermo Fisher Scientific, Waltham, MA, USA) according to the manufacturer’s instructions. An external control miRNA, cel-miR-39-3p from Caenorhabditis elegans, was added to each sample at the start of the procedure to ensure reliable and efficient miRNA isolation. A fixed amount of cel-miR-39-3p was spiked into each plasma sample prior to extraction. The quality and quantity of total RNA were assessed using the Take3 plate on a Synergy H1M1 BioTek reader (BioTek Instruments, Winooski, VT, USA).

Tissue samples were homogenized using metal beads and lysis buffer with a Roche MagNA Lyser Benchtop Homogenizer (Roche Holding, Basel, Switzerland). Total RNA was isolated using the AllPrep DNA/RNA/Protein Kit (QIAGEN, Hilden, Germany) according to the manufacturer’s instructions. The quality and quantity of the isolated RNA were also evaluated using the Take3 plate on the Synergy H1M1 BioTek platform.

### 2.4. Reverse Transcription and Real-Time Polymerase Chain Reaction

miRNA detection was performed using the TaqMan™ MicroRNA Assay system (Thermo Fisher Scientific, Waltham, MA, USA). Candidate miRNAs were selected based on bioinformatic analysis and literature evidence, showing a prominent role in the pathways of interest. The top miRNAs were chosen for their regulation of the highest number of shared targets identified through the DisGeNET derived shared gene set and for regulating genes associated with at least five enriched biological terms (focused on that which play an important role in p53 regulation, i.e., PINK, PTEN, for details see Table 1) and metabolites—miR-16-5p; miR-34a-5p; miR-124-3p and miR-548c-3p. Two additional miRNAs were chosen according to the literature data, as they had been reported to regulate neurotrophic processes and glioma-related genes (miR-29a-3p [26,27] and miR-32-5p [28]). Thus, six miRNAs were selected in total; due to plasma detectability constraints, four miRNAs were quantified in plasma and six in brain tissue. In total, plasma was screened for four miRNAs (miR-16-5p, miR-29a-3p, miR-32-5p, miR-34a-5p) and two controls (cel-miR-39-3p and U6 snRNA), and brain tissue for six miRNAs (miR-16-5p, miR-32-5p, miR-29a-3p, miR-34a-5p, miR-124-3p, miR-548c-3p) and the control U6 snRNA. miR-124-3p and miR-548c-3p were undetectable in plasma (Ct > 40). For plasma, cel-miR-39-3p was used to assess extraction/RT performance (quality control), whereas relative miRNA expression was normalized to U6 snRNA (endogenous reference) unless stated otherwise. For tissue, relative miRNA expression was normalized to U6 snRNA.

Reverse transcription was carried out using the TaqMan™ MicroRNA Reverse Transcription Kit (Catalog number 4366596; Thermo Fisher Scientific, Waltham, MA, USA). Each reaction mixture consisted of 5 μL of totalRNA (containing 5 ng of totalRNA), 7 μL of reaction mix (100 mM dNTPs, MultiScribe reverse transcriptase, 10 × reverse transcription buffer, RNase inhibitor and nuclease-free water) and 3 μL of RT primer. Reverse transcription was performed using the following thermocycler protocol: 16 °C for 30 min, 42 °C for 30 min, and 85 °C for 5 min. After the reaction, samples were stored at −20 °C until further processing. The samples were submitted to an amplification procedure. One reaction contained 10 μL of TaqMan Fast Advanced Master Mix (Catalog number 4444556), 7.67 μL of nuclease free water, 1 μL of TaqMan MicroRNA Assay (probe; miR-16-5p, Catalog number 000391; miR-32-5p, Catalog number 002109; miR-29a-3p, Catalog number 002112; miR-34a-5p, Catalog number 000426; miR-124-3p, Catalog number 003188_mat; miR-548c-3p, Catalog number 001590; cel-miR-39, Catalog number 000200; U6 snRNA, Catalog number 001973) and 1.33 μL of cDNA. The thermocycler protocol was as follows: 95 °C for 20 s and 45 cycles of 95 °C for 1 s and 60 °C for 20 s. The amplification was performed on a RotorGene Q 5plex HRM System (QIAGEN, Hilden, Germany). To minimize variability in RNA extraction and reverse transcription among samples, the U6 small RNA was used as a control for each sample. The relative expression levels of miRNAs were calculated using the efficiency-adjusted real-time PCR method [29].

### 2.5. ELISA

As the p53 protein has a well-established role as a key tumor suppressor involved in cell cycle regulation, apoptosis, and DNA repair, and its altered expression is commonly associated with glioma progression, we quantified p53 levels in tumor and peritumoral tissues to explore potential associations with the selected miRNAs. The protein concentrations of p53 in tumor and peritumoral tissues were determined using a commercially available ELISA kit—p53 protein kit Cusabio, with a range of detection 9.38 pg/mL—600 pg/mL (Catalog number CSB-E08334h). Analysis of all samples was performed according to the manufacturer’s protocol.

Frozen tissue was thawed on ice, fragmented and sonicated for 30 s with PBS (1:9 ratio) and a protease inhibitor (5 μL per 100 mg of tissue). The homogenates were centrifuged (5 min; 4 °C, 5000× *g*), collected in new tubes, aliquoted and frozen at −70 °C until further analyzed. The optical density was determined using a microplate reader (Synergy H1, BioTek) set to 450 nm. The total protein content in each sample was examined based on the Pierce method (PierceTM 660 nm Protein Assay, Thermo Scientific, Catalog number 22662). The results are expressed as pg/ng of the total protein.

### 2.6. Statistical Analysis

Plasma and brain levels of tested miRNAs and p53 are presented using box plots, whose limits represent the 25th and 75th percentiles as determined by R software (2026) whiskers extend 1.5 times the interquartile range from the 25th and 75th percentiles; outliers are represented by dots. Center lines show the medians and cross the means. Analysis of variance (ANOVA) followed by the least significant difference (LSD) test was used to assess differences in the miRNA relative expression in plasma between patients with discopathy, PD and gliomas. A t-test was used to compare tumor and peritumoral tissue expression of miRNA and p53. All statistical analyses were performed using StatSoft Statistica 13 (2016, USA). For enrichment analysis, an adjusted *p*-value (*p*-adj) < 0.05 was used as the significance threshold. No formal adjustment for multiple testing was applied to the plasma and tissue expression analyses.

## 3. Results

### 3.1. Initial Bioinformatic Assessment

#### 3.1.1. Gene List Extraction

A total of 953 genes shared between PD and glioblastoma were identified using the DisGeNET database. DisGeNET integrates data from multiple sources, including curated repositories (e.g., UniProt, ClinVar), genome-wide association studies (GWAS), animal models, and the scientific literature. Each gene–disease association is assigned a confidence score reflecting the level of evidence and frequency of co-mention across publications and datasets. In this study, these shared genes represent the literature-based disease-associated genes, not differentially expressed genes from transcriptomic datasets.

**Figure 2:**
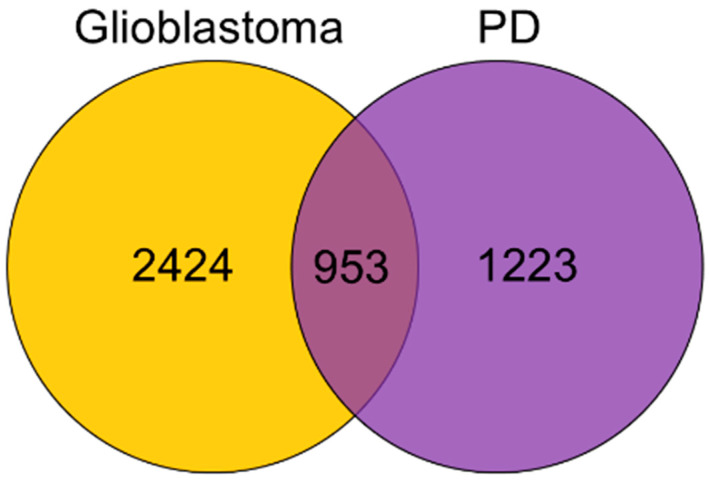
Overlap between genes associated with glioblastoma and PD according to DisGeNET database.

Only genes with associations supported by evidence from human studies and curated sources were selected to ensure reliability. This integrative approach allowed us to compile a robust list of genes potentially implicated in the overlapping molecular pathology of PD and glioblastoma. Figure 2 provides a simplified overview of the results of this gene extraction analysis.

#### 3.1.2. miRNA Ranking

Table 1 shows the results of the ranking analysis based on the number of predicted targets among the genes shared between both diseases, with the top five miRNAs identified: miR-16-5p, miR-548c-3p, miR-124-3p, miR-1-3p, and miR-34a-5p. Among these, four miRNAs (miR-16-5p, miR-548c-3p, miR-124-3p and miR-34a-5p) were selected for experimental validation based on their high ranking and functional relevance. Those results were crucial for our further molecular analysis, as those miRNAs were chosen to be amplified in the biological material collected during the study.

Two of the miRNAs were chosen according to the literature data, based on reports indicating their involvement in neurotrophic and glioma-related processes (miR-29a-3p [26,27] and miR-32-5p [28]). The complete list of predicted targets for each candidate miRNA in the shared PD-glioblastoma gene set, as well as in the glioblastoma-only and PD-only gene sets, is provided in Supplementary Table S1 (gene-level lists per miRNA and gene set).

### 3.2. Molecular Assessment

#### 3.2.1. miRNAs Expression in the Plasma of Patients with Discopathy, PD and Glioblastoma

**Figure 3:**
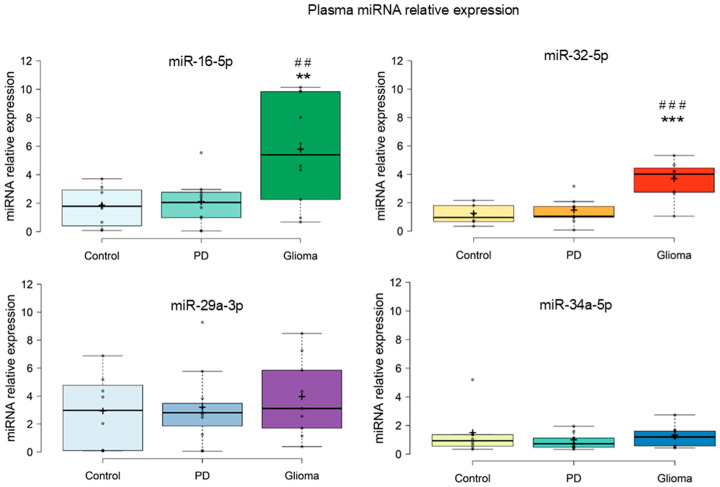
Relative expression of miR-16-5p, miR-32-5p (top panels), miR-34a-5p and miR-29a-3p (bottom panels) in the plasma of patients with discopathy (control), PD and glioma. Center lines show the medians; box limits indicate the 25th and 75th percentiles as determined by R software; whiskers extend 1.5 times the interquartile range from the 25th and 75th percentiles; outliers are represented by dots; crosses represent sample means; data points are plotted as open circles. Group differences were assessed using one-way ANOVA followed by least significant difference (LSD) post hoc tests. (**, ##—indicates significant differences vs. control; *p* < 0.01, ***, ###—indicate significant differences vs. PD; *p* < 0.001).

Figure 3 presents the relative plasma expression of miR-16-5p, miR-32-5p, miR-29a-3p, and miR-34a-5p in the control group (discopathy), PD patients, and patients with gliomas. ANOVA showed significant changes in the level of miR-16-5p and miR-32-5p between the three studied groups of patients (F_miR-16-5p_ = 7.26; P_miR-16-5p_ = 0.003; F_miR-32-5p_ = 12.79; P_miR-32-5p_ = 0.0003). Increased levels of plasma miR-16-5p and miR-32-5p in patients with gliomas, compared to patients with discopathy (P_miR-16-5p_ = 0.003; P_miR-32-5p_ = 0.0003) and PD (P_miR-16-5p_ = 0.003; P_miR-32-5p_ = 0.0003) were observed. No changes in the level of plasma miR-16-5p (*p* = 0.84) and miR-32-5p (*p* = 0.67) were observed between the patients with discopathy and PD (Figure 3). The relative expression of miR-16-5p (normalized to the housekeeping gene) was 5.7 in plasma from patients with gliomas, 2.0 in plasma from PD patients, and 1.76 in plasma from patients with discopathy. This corresponds to approximately a 2.85-fold increase in plasma miR-16-5p expression in patients with gliomas compared to those with PD and a 3.24-fold increase compared to patients with discopathy. The relative expression of miR-32-5p (normalized to the housekeeping gene) was 3.6 in plasma from patients with gliomas, 1.4 in plasma from PD patients, and 1.1 in plasma from patients with discopathy. This corresponds to approximately a 2.0-fold increase in plasma miR-32-5p expression in glioma patients compared to those with PD, and a 3.2-fold increase compared to patients with discopathy.

ANOVA showed no changes in the levels of miR-29a-3p (F = 0.35; *p* = 0.71) and miR-34a-5p (F = 0.63; *p* = 0.54) in plasma between patients with discopathy, PD and glioma (Figure 3). Among all measured plasma miRNAs, only miR-16-5p and miR-32-5p showed significant differences between groups.

#### 3.2.2. miRNAs Expression in Tumor and Peritumoral Tissue

**Figure 4:**
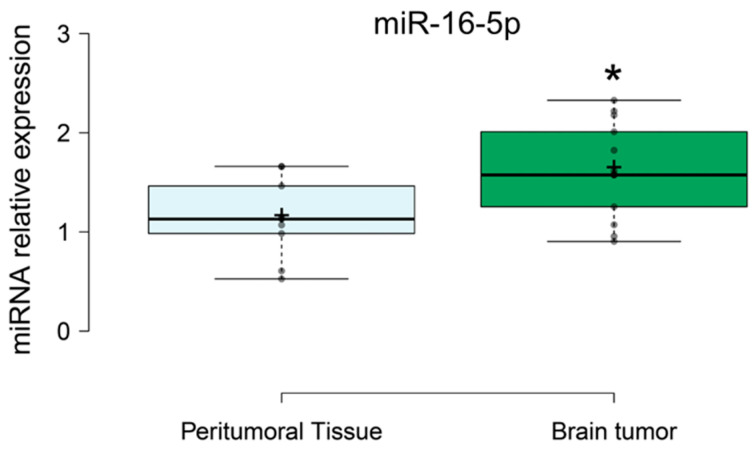
Relative expression of miR-16-5p in peritumoral and tumoral tissue. Center lines show the medians; box limits indicate the 25th and 75th percentiles as determined by R software; whiskers extend 1.5 times the interquartile range from the 25th and 75th percentiles; outliers are represented by dots; crosses represent sample means; data points are plotted as open circles. * *p* < 0.05.

Figure 4 shows the relative expression of miR-16-5p in peritumoral and tumoral tissue. A t-test showed differences in the level of tissue miR-16-5p between tumoral and peri-tumoral tissue (t = 2.46745) and revealed no differences in the level of the rest miRNAs tested (miR-32-5p, miR-34a-5p; miR-29a-3p, miR-124-3p, and miR-548c-3p). miR-16-5p was increased in tumor tissue compared to peritumoral tissue (*p* = 0.023). The relative expression of miR-16-5p (normalized to the housekeeping gene) was 1.6 in tumoral tissue and 1.1 in peritumoral tissue. This corresponds to approximately a 1.45-fold increase in miR-16-5p expression in tumoral compared to peritumoral tissue (Figure 4). For tissue samples, none of the other analyzed miRNAs showed significant differences between tumor and peritumoral tissue.

#### 3.2.3. Tumor Protein p53 (p53) Expression in Tumor and Peritumoral Tissue

**Figure 5:**
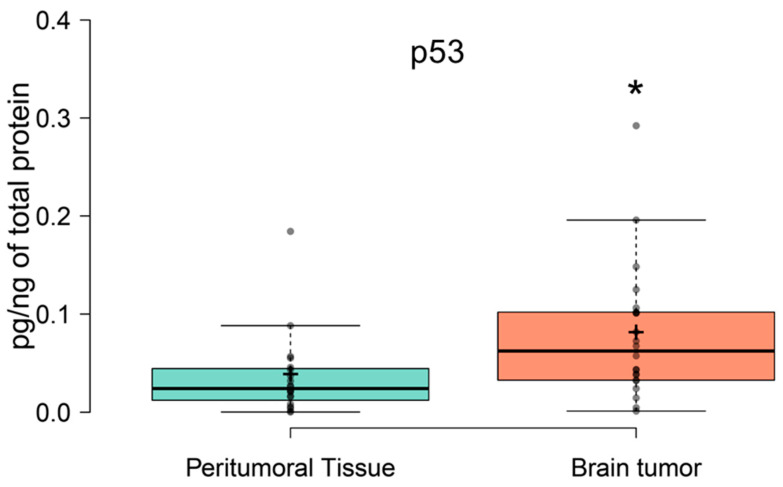
p53 expression (pg/ng of total protein) in the peritumoral and tumor tissue. Center lines show the medians; box limits indicate the 25th and 75th percentiles as determined by R software; whiskers extend 1.5 times the interquartile range from the 25th and 75th percentiles; outliers are represented by dots; crosses represent sample means; data points are plotted as open circles. * *p* < 0.05.

Figure 5 presents p53 expression (pg), normalized to total protein, in peritumoral and tumor tissue. A t-test showed differences in the level of tissue p53 between tumoral and peritumoral tissue (t = 2.423932). p53 concentration was increased in tumor tissue compared to peritumoral tissue (*p* = 0.02). The mean p53 concentration in tumor tissue was 0.08 pg/ng of protein and in peritumoral tissue it was 0.036 pg/ng of protein. This corresponds to approximately a 2.2-fold increase in p53 concentration in tumoral compared to peritumoral tissue and reflects the well-established role of the p53 protein in glioma biology (Figure 5).

### 3.3. Final Bioinformatic Assessment

#### Final Enrichment Analysis for miR-16-5p and miR-32-5p

**Figure 6:**
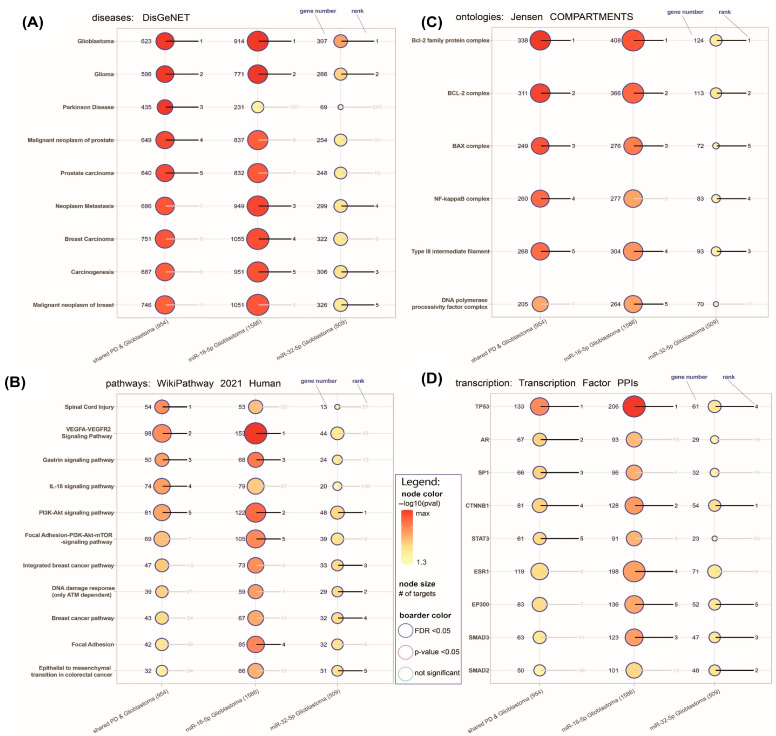
Enriched terms shared between common genes for miR-16-5p and miR-32-5p, and enriched in those miRNAs (**A**) diseases, (**B**) pathways, (**C**) protein complexes, (**D**) transcription Factors of genes shared between miR-16-5p and miR-32-5p. The adjusted *p*-values show categories that are more likely to have biological meanings. Significant terms after adjustment are marked with a blue circular border. The color gradient of the dots is associated with corresponding adjusted *p*-values. Red color indicates low *p*-values (high enrichment) and yellow indicates high *p*-values (low enrichment). The size of the dots is associated with the number of enriched genes. Numbers on the left side of the dots are gene numbers, on the right side are enrichment ranks (lower better). # number of targets.

Figure 6 presents the results of final enrichment analysis for miR-16-5p and miR-32-5p, in the context of diseases, pathways, protein complexes and transcription factors. To elucidate the functional context of the shared target genes and miR-16-5p and miR-32-5p, a multi-layered enrichment analysis (adjusted *p*-value < 0.05) was performed across disease associations, signaling pathways, protein complexes, and transcription factor interactions. Enrichment analysis offers a powerful means to contextualize gene lists by identifying overrepresented biological themes; in this study, analyses were performed using the Enrichr database, which integrates diverse curated sources (e.g., DisGeNET, WikiPathways, Jensen, and TF-PPI) and calculates statistical significance based on gene set enrichment using Fisher’s exact test followed by multiple testing correction. This analysis aimed to identify convergent biological processes and molecular mechanisms potentially co-regulated by both miRNAs. The results revealed significant enrichment in diseases such as glioblastoma, glioma, and PD (DisGeNET), suggesting potential shared involvement in neurooncological and neurodegenerative mechanisms. Pathway analysis (WikiPathways) highlighted signaling cascades related to Spinal Cord Injury, VEGFA-VEGFR2 signaling (vascular endothelial growth factor, vascular endothelial growth factor receptor 2), gastrin signaling, interleukin 18 (IL-18), and PI3K-Akt signaling (phosphoinositide 3-kinase signaling), indicating convergence on inflammation, angiogenesis, and neuroplasticity-related processes. Enrichment in protein complexes (Jensen COMPARTMENTS), including the B-cell lymphoma-2 family (BCL-2 family), BCL-2 complex, BCL-2 Associated X complex (BAX complex), and nuclear factor kappa-light-chain enhancer of activated B cells complex (NFKB1), further points to regulatory roles in apoptosis and inflammation. Finally, transcription factor interaction analysis (Transcription Factor PPI) indicated associations with tumor protein 53 (TP53), androgen receptor (AR), Specificity Protein 1 (SP1), and catenin Beta 1 (CTNNB1), suggesting transcriptional coregulation of stress- and cancer-related pathways.

**Figure 7:**
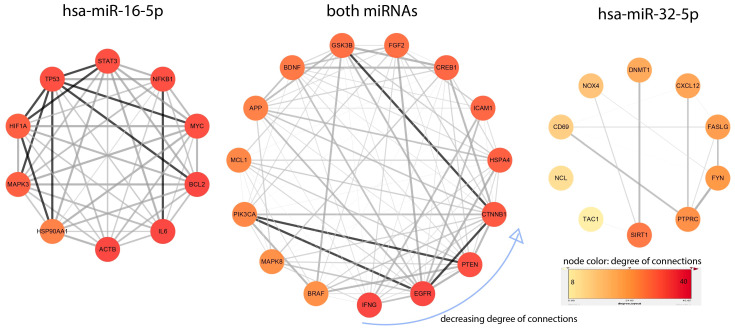
Interaction networks of genes shared between PD and glioblastoma for miR-16-p, both miRNAs and miR-32-5p. Interactions between networks are hidden. Interaction networks were arranged in a circular layout, ordered by decreasing degree of connections. Interactions between networks are hidden. The gradient of node color, from dark red (highly connected) to yellow (low connectivity), indicates the degree of interaction centrality within each miRNA-specific or shared network.

Figure 7 presents the result of interaction network analysis performed to identify top genes regulated by miR-16-5p, miR-32-5p and shared between both miRNAs. Interaction network analysis was conducted to delineate the top-ranking gene interactors regulated by miR-16-5p, miR-32-5p, and those shared between both miRNAs, focusing on genes implicated in both PD and glioblastoma. In the miR-16-5p-specific network, key hub genes included actin beta (ACTB), interleukin 6 (IL-6), and BCL2, which are known to play pivotal roles in cytoskeletal organization, inflammation, and apoptosis, respectively. The network also included TP53, signal transducer and activator of transcription 3 (STAT3), oncogene myelocytoma (MYC), and NFKB1, further emphasizing the link to cancer-related signaling and immune regulation.

For miR-32-5p, the top interactors were sirtuin1 (SIRT1), a key regulator of mitochondrial function and cellular stress responses; protein tyrosine phosphatase receptor type C (PTPRC), a marker of immune activation; and FYN proto-oncogene (FYN), involved in neuroinflammation and synaptic plasticity. The lower degree of connectivity in this network, as shown by lighter node colors, indicates a more selective regulatory role compared to miR-16-5p.

The shared miRNA network (middle panel) revealed interferon gamma (IFNG), epidermal growth factor receptor gene (EGFR), and phosphatase and TENsin homolog (PTEN) as central nodes. These genes are involved in immune signaling (IFNG), cell proliferation and migration (EGFR), and tumor suppression (PTEN). Additional shared hubs such as brain-derived neurotrophic factor (BDNF), mitogen-activated protein kinase 8 (MAPK8), phosphatidylinositol-4,5-bisphosphate 3-kinase, catalytic subunit alpha (PIK3CA), and cadherin-associated protein beta (CTNNB1) suggest convergence on pathways regulating neurotrophic signaling, apoptosis, and PI3K-Akt/mammalian/mechanistic target of rapamycin (mTOR) cascades, reinforcing the relevance of these genes in both neurodegenerative and oncogenic contexts. Notably, the shared network exhibited higher overall connectivity, suggesting that common targets of miR-16-5p and miR-32-5p may act as regulatory bottlenecks at the interface of PD and glioma pathogenesis. These results underscore the potential of these miRNAs to modulate overlapping molecular mechanisms across neurodegeneration and tumor progression.

## 4. Discussion

In our study, we aimed to characterize the molecular basis of the clinical observation of the inverse correlation between the incidence of gliomas and neurodegenerative disorders, specifically PD. Epidemiological studies suggest an inverse comorbidity between PD and several cancers. This observation motivated our molecular analysis of shared pathways in both conditions, with a focus on TP53, PI3K-Akt, and NFKB1, which link glioblastoma biology with PD-related disturbances [30,31].

Using bioinformatic tools, we identified 953 genes shared between glioblastoma and PD (Figure 2). We also ranked the top miRNAs (miR-16-5p, miR-548c-3p, miR-124-3p, miR-1-3p, and miR-34a-5p, Table 1) that were, with miRNAs chosen on the basis of the literature data (miR-29a-3p and miR-32-5p), used for further molecular analyses in clinical samples. Our findings demonstrated elevated levels of miR-16-5p and miR-32-5p in the plasma of glioma patients compared to patients with PD and control (discopathy). Furthermore, miR-16-5p expression was significantly increased in glioma tumor tissue compared to peritumoral tissue, alongside elevated levels of the p53 protein. Enrichment analysis for miR-16-5p and miR-32-5p revealed shared diseases—glioblastoma, glioma and Parkinson’s disease; pathways—such as spinal cord injury, VEGFA-VEGFR2 signaling, gastrin signaling, IL-18 and PI3K-Akt signaling; protein complexes—including BCL-2 family, BCL-2 complex, BAX complex and NFKB1 complex; and transcription factors—TP53, AR, SP1, and CTNNB1 (Figure 6). Taken together, these multi-layered results point to overlapping regulatory circuits potentially influenced by miR-16-5p and miR-32-5p at the interface of neurodegeneration and tumorigenesis.

In recent years, miRNAs have gained significant attention in the search for reliable, non-invasive, and cost-effective peripheral biomarkers for the diagnosis of cancer and other diseases [32]. Their ability to cross the blood–brain barrier (through EVs), which increase miRNAs’ stability and biological activity [33,34], stability in peripheral blood, tissue specificity, and ease of detection position miRNAs as leading candidates for potential peripheral biomarkers, which could be implemented in clinical practice as part of a diagnostic panel. Consistent with this concept, our findings demonstrated elevated levels of miR-16-5p and miR-32-5p in the plasma of glioma patients compared to patients with PD and control (discopathy) (Figure 3). These results align with the growing field of glioma research focused on identifying circulating biomarkers, in which circulating miRNAs, including members of the miR-16 family, have demonstrated promising diagnostic potential [35,36]. miR-16-5p regulates the largest number of genes in glioblastoma and PD (it was the highest-ranked miRNA in our ranking analysis) and is associated with the regulation of cell cycle and neurotrophic processes [37,38]. Its higher plasma levels in patients with glioma may reflect extensive dysregulation of tumor cell proliferation, but also may mirror an EV-mediated role as a peripheral messenger involved in metastasis, activation of compensatory mechanisms, or initiation of stress responses [27,33,34,39,40].

The role of miR-32-5p in gliomas is also well established [28,41] and several studies have indicated its tumor-suppressive function. One of the proposed mechanisms by which it inhibits cell proliferation and migration involves direct targeting of the EZH2 gene (Enhancer of Zeste Homolog 2) [41], an oncogene. In our study, we reported no changes in the expression of miR-32-5p in peritumoral compared to tumoral tissue; however, we found increased levels of this molecule in the plasma of glioma patients compared to PD and control patients. We therefore suggest a selective release mechanism of miRNA-32-5p, rather than a simple reflection of tumor abundance. A possible explanation involves EV-mediated export of miRNA-32-5p from tumor cells as a part of a coordinated process counteracting its tumor-suppressive activity [42]. Such a selective mechanism explains why peripheral levels of miRNA-32-5p are increased when tissue levels remain stable or even decreased and supports miRNA-32-5p as a potential plasma biomarker of glioma activity. This interpretation remains to be tested directly in functional experiments but is consistent with current knowledge on EV-mediated miRNA export.

In our study, we observed increased levels of miR-16-5p in tumoral tissue compared to peritumoral (adjacent) tissue (Figure 4). Similarly, elevated concentrations of miR-16-5p have been reported in tissues from patients with breast cancer, colorectal cancer, and malignant mesothelioma [43,44,45,46]. However, contradictory data also exist, with studies reporting decreased miR-16-5p levels in tumor tissues compared to adjacent tissue [40,47]. These discrepancies highlight the complex, context-dependent role of miR-16-5p in cancer-related processes. While its inhibitory effect on cell proliferation is well documented, the specific regulatory mechanisms underlying its expression remain unclear and may vary based on factors such as cancer type or disease stage. In vitro studies have shown that miR-16-5p inhibits glioma cell migration and invasion by targeting the BCL-2 gene [48] and sal-like protein 4 (SALL4) [36]. This inhibitory potential, observed in other types of cancers, may also involve cooperation with other miRNAs, such as those in the miR-15/16 cluster, a known tumor suppressor that regulates intracellular pathways mediated by BCL2, MCL1, CCND1, and Wnt Family Member 3A (WNT3A) [48]. Additionally, this association of miR-16-5p may involve direct impact on oncogenes such as MYC-associated neuroblastoma (MYCN) [37]. Increased expression of miR-16-5p in cancer tissue may serve as a strategy to induce tumor cell death, result from dysregulated intracellular signaling pathways involving miR-16-5p, or arise from epigenetic dysregulation, such as altered DNA methylation affecting miRNA clusters [40,49,50].

The increased level of miR-16-5p in tumor tissue was accompanied by upregulated expression of the p53 protein (Figure 5). Moreover, our final enrichment analysis identified TP53 (the gene encoding p53) as the top transcription factor predicted to interact with miR-16-5p (Figure 6). The p53 protein plays a pivotal role in essential cellular processes, including DNA repair, regulation of the cell cycle, and apoptosis. Loss of its function disrupts the mechanisms controlling cell proliferation and survival, thereby contributing to cancer development. The interaction between miR-16-5p and p53 has been the focus of intensified research in recent years [51,52]. It has been found that miR-16-5p targets and decreases sestrin 1 (SESN1) expression, leading to reduced proliferation, enhanced apoptosis, and inhibited differentiation of myoblasts. Furthermore, SESN1 has been shown to be involved in the regulation of p53 signaling pathways [46]. In breast cancer, p53 represses the expression of two kinases, checkpoint kinase 1 (Chk1) and Wee1 G2 checkpoint kinase (Wee1), through the upregulation of miR-16 and miR-26a, which is believed to sensitize cancer cells to genotoxic therapy [51]. In glioma cells overexpressing miR-16-5p, an increased level of p53 has also been observed [52].

These findings are consistent with a possible functional relationship between miR-16-5p and p53, but do not establish the directionality or mechanism of this association. However, whether this interaction is universally beneficial or context-dependent requires further validation. A bidirectional model is plausible: p53 can raise miR-16 family activity, while miR-16-5p limits pro-survival effectors (e.g., BCL2/MCL1), together making glioma cells more ready to undergo apoptosis. Our data support this model at the level of correlative expression, but mechanistic studies will be required to confirm a causal relationship. The inclusion of p53 in the present study was not intended to establish it as a novel glioma biomarker, since its upregulation in malignant gliomas has been widely reported. Rather, p53 was analyzed as a molecular marker within the biological framework of this study, which focused on pathways linking proliferation, apoptosis, and stress responses to the opposing processes of tumorigenesis and neurodegeneration. Accordingly, the p53 data should be interpreted as supportive rather than as a central or independent finding of the present study.

We subsequently performed a targeted bioinformatic analysis of these two differentially expressed miRNAs. The enrichment analysis identified the following shared elements: diseases—glioma, glioblastoma, PD, malignant neoplasm of prostate, and prostate carcinoma; pathways—spinal cord injury, VEGFA-VEGFR2 signaling, gastrin signaling, IL-18 signaling, and PI3K-Akt signaling pathways; protein complexes—BCL2, BCL2-family complexes, BAX complex, NFKB1, type III intermediate filament; and transcription factors—TP53, AR, SP1, CTNNB1, and STAT3 (Figure 6). Functionally, these pathways meet in growth–survival and stress-response programs. In this context, higher tissue miR-16-5p with higher p53 may be interpreted as compatible with altered apoptosis- and stress-response signaling, while higher plasma miR-32-5p may be consistent with EV-mediated export linked to PI3K-Akt/SOX activity in the tumor microenvironment and may reflect one of several possible mechanisms of release into the circulation, including but not limited to extracellular vesicle-associated export. These findings should be interpreted cautiously but reinforce the potential of miR-16-5p and miR-32-5p as peripheral biomarkers and highlight their involvement in pathways critical to both glioma and PD.

miR-16-5p and miR-32-5p have been identified as promising peripheral biomarkers for gliomas. According to our molecular analysis and bioinformatic results, these miRNAs are associated with the glioma-related pathways, offering significant potential for further correlation with clinical symptoms. However, additional studies involving a larger cohort of patients are required to validate and expand upon our hypothesis.

Unfortunately, despite extensive initial bioinformatic assessment, none of the top-ranked miRNAs have been found to be differentially expressed in PD when compared to controls (discopathy). However, it cannot be excluded that the identified miRNAs, pathways, and processes are involved in the inverse correlation between glioma and PD incidence. This is supported by the following facts: 1. the lower levels of miR-16-5p and miR-32-5p in PD compared to gliomas, 2. the enrichment analysis (for miR-16-5p and miR-32-5p) showing top shared diseases, which include gliomas and PD, and 3. enrichment analysis (for miR-16-5p and miR-32-5p) of shared pathways that include the PI3K pathway. At the current stage, these observations should be viewed as suggestive of shared biology rather than as evidence for altered PD risk.

Taken together, our data should be regarded as hypothesis-generating rather than definitive. The statistically significant differences in miR-16-5p, miR-32-5p and p53 are accompanied by overlapping distributions between groups, reflecting the small sample size and the exploratory nature of this study. As a consequence, the possible contribution of these miRNAs to the inverse epidemiological association between PD and glioma requires confirmation in larger cohorts and functional models.

While our findings shed light on the molecular overlap between glioma and PD, several limitations must be addressed. First, the small sample size may have limited the statistical power to detect subtle differences in miRNA expression. Second, our focus on peripheral blood and tumor tissue does not fully capture the dynamic interactions within the CNS. Third, the bioinformatic layer relies on disease–gene associations and predicted miRNA–target interactions of varying strength and should be supplemented with analysis of direct expression or functional validation in larger cohorts of patients. Future studies should explore the functional role of miR-16-5p and miR-32-5p in preclinical models, as well as their interactions with key pathways such as PI3K-Akt and p53 signaling.

Finally, the observed inverse correlation between glioma and PD requires further investigation. While lower miR-16-5p and miR-32-5p levels in PD compared to gliomas are compatible with this hypothesis, direct mechanistic evidence linking these miRNAs to disease incidence remains elusive. High-throughput screening and longitudinal studies will be crucial in elucidating whether shared molecular pathways underlie this phenomenon or if it results from unrelated systemic factors. Taken together, our tissue (miR-16-5p ↑/p53 ↑) and plasma (miR-16-5p ↑, miR-32-5p ↑) results give a miRNA basis for non-invasive glioma assessment, which now needs prospective testing and comparison with standard glioblastoma miRNAs. Our data support a coherent, strand-resolved working model in which miR-16-5p and miR-32-5p capture complementary facets of glioma biology: within tumor tissue, miR-16-5p co-elevates with p53 and constrains pro-survival circuitry (e.g., BCL2/MCL1, CCND1), indicating engagement of an apoptosis-competent, stress-response axis; in the circulation, higher miR-32-5p most plausibly reflects selective extracellular-vesicle export of a tumor-suppressive molecule. Convergence of both miRNAs on TP53, PI3K-Akt and NFKB1 pathways provides a mechanistic bridge to our enrichment results and may help to explain the disease contrast with PD, where the proliferative/angiogenic context and EV traffic are attenuated. Practically, a small, strand-aware panel combining miR-16-5p (tissue/plasma) with miR-32-5p (plasma) offers a biologically grounded framework for non-invasive assessment and hypothesis-driven stratification (apoptosis competence, PI3K/SOX dependency) that now needs to be prospectively validated.

Our study presents a preliminary approach, and we are aware that this area should be further explored, especially using high-throughput methods such as single-cell sequencing. Therefore, we recognize several limitations of our study. First, the research was performed using biological material collected from a single center. In addition, the study groups differed in age and sex distribution, the glioma cohort included both grade III and grade IV tumors, and the PD cohort represented advanced patients selected for DBS, which limits the generalizability of the findings and may have introduced cohort-related bias.

## 5. Conclusions

miR-16-5p and miR-32-5p have been identified as potential peripheral biomarkers of gliomas. Further studies involving larger patient cohorts are necessary to validate and expand upon this hypothesis. A properly designed study would help to determine whether the peripheral levels of miR-16-5p and miR-32-5p correlate with clinical symptoms, disease progression, or response to pharmacological treatment. Although no significant changes in the expression of miR-16-5p and miR-32-5p were observed in PD compared to discopathy (control), their potential involvement in the molecular basis underlying the inverse correlation between glioma incidence and PD cannot be entirely excluded. This is supported by the differential expression of these miRNAs in PD versus gliomas, as well as bioinformatic results indicating overlapping intracellular signaling pathways, including shared protein complexes and transcription factors, between the two diseases. These observations should be interpreted in the context of the small, single-center, clinically heterogeneous cohorts analyzed in this study. Thus, the present data do not allow conclusions about causal effects on PD risk. To explore this phenomenon in greater depth, further investigations are warranted. These should include studies using cell lines and preclinical models of both PD and gliomas, as well as larger-scale clinical studies employing high-throughput screening methods.

## Figures and Tables

**Table 1:** In silico prediction of the top miRNAs regulating shared genes between PD and glioblastoma.

Predicted miR	Number of Targeted Genes					Target Symbols for Selected Pathways (Gene Number)				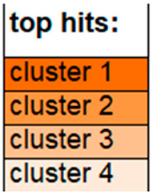
Mature Mirna Id	Shared Genes (954)	Glioblastoma (3377)	Parkinson Disease (2177)	Wikipathway Pi3k-Akt Signaling Pathway (82)	Top Metabolites (93)	PTEN/PI3K-AKT-mTOR (9)	PTEN/PINK1-PARK6-parkin (5)	LRKK2-PI3K-AKT-mTOR (8)	WNT-β-catenin-pathway-p-53 (5)	
hsa-miR-16-5p	483	1588	1014	56	27	MTOR|PIK3CA|PTEN	PINK1|PTEN	MTOR	CTNNB1|TP53|WNT3A	
hsa-miR-548c-3p	414	1424	882	51	22	AKT1|MTOR|PIK3C3|PIK3CA|PTEN	PINK1|PTEN	MTOR|PIK3C3	CTNNB1	
hsa-miR-124-3p	449	1590	949	49	29	AKT1|MTOR|PIK3CA	PINK1	MTOR	CTNNB1	
hsa-miR-1-3p	470	1604	1027	46	26	AKT1|PIK3C3|PIK3CA|PTEN	PTEN	PIK3C3	CTNNB1|TP53	
hsa-miR-34a-5p	415	1364	821	44	25	AKT1|PIK3CA|PTEN	PINK1|PTEN		CTNNB1|TP53|WNT3A	
hsa-miR-155-5p	422	1331	841	51	21	AKT1|PIK3CA|PTEN	PTEN		CTNNB1|TP53	
hsa-miR-27a-3p	408	1376	868	53	16	AKT1|MTOR|PIK3C3|PIK3CA	PINK1|PRKN	MTOR|PIK3C3	CTNNB1|TP53|WNT3A	
hsa-miR-107	369	1240	760	42	14	PTEN	PINK1|PTEN		CTNNB1|TP53|WNT3A	
hsa-let-7b-5p	379	1295	770	42	17	AKT1|MTOR|PIK3CA	PINK1	MTOR	TP53	
hsa-miR-590-3p	375	1259	800	53	14	PIK3C3|PIK3CA|PTEN	PTEN	PIK3C3		
hsa-miR-23b-3p	365	1143	737	38	13	PIK3C3|PTEN	PTEN	PIK3C3	CTNNB1|TP53	
hsa-miR-3163	353	1231	758	45	13	PIK3C3|PIK3CA|PTEN	PTEN	PIK3C3	TP53	
hsa-miR-374a-5p	338	1126	669	38	20	AKT1|PIK3C3|PIK3CA|PTEN	PTEN	PIK3C3	CTNNB1	
hsa-miR-186-5p	358	1211	745	45	17	PIK3C3|PIK3CA|PTEN	PTEN	PIK3C3	TP53|WNT3A	

Data was analyzed using OneR algorithm implemented in the wizbionet package vignette-wizbionet; author Zofia Wicik; date 2 September 2020. Using this machine-learning methodology, miRNAs are grouped into four clusters based on the number of predicted targets in shared and disease-specific gene sets. The background color indicates the OneR cluster assignment (cluster 1—dark orange, cluster 2—orange, cluster 3—bright orange, cluster 4—pale orange). In parentheses are the numbers of mapped genes from each gene list.

## Data Availability

Data supporting the findings of this study are available from the corresponding author upon request. The results of the bioinformatic analyses are provided in Supplementary Table S1.

## Supplementary Materials

The following supporting information can be downloaded at: https://www.mdpi.com/article/10.3390/brainsci16040347/s1, (Supplementary Table S1—gene-level lists per miRNA and gene set).
